# Analytical Biomarkers for Inflammation Status Monitoring of Psychotropic and Antiepileptic Drugs

**DOI:** 10.3390/ph18081213

**Published:** 2025-08-17

**Authors:** Wiktoria Jiers, Karina Sommerfeld-Klatta, Mehmet Gumustas, Paul Mozdziak, Magdalena Łukasik-Głębocka, Artur Teżyk, Zbigniew Żaba, Czesław Żaba, Hanna Piotrowska-Kempisty

**Affiliations:** 1 Department of Toxicology, Poznań University of Medical Sciences, 3 Rokietnicka Street, 60-631 Poznań, Poland; ksommerfeld@ump.edu.pl (K.S.-K.); hpiotrow@ump.edu.pl (H.P.-K.); 2 Doctoral School, Poznań University of Medical Sciences, 70 Bukowska Street, 60-812 Poznań, Poland; 3 Institute of Forensic Sciences, Department of Forensic Toxicology, Ankara University, Balkiraz, Mamak Cd.6 A, Mamak, Ankara 06620, Turkey; mgumustas@ankara.edu.tr; 4 Prestige Department of Poultry Science, College of Agriculture and Life Sciences, North Carolina State University, Raleigh, NC 27695, USA; pemozdzi@ncsu.edu; 5 Department of Emergency Medicine, Poznań University of Medical Sciences, 7 Rokietnicka Street, 60-806 Poznań, Poland; magdaglebocka@ump.edu.pl (M.Ł.-G.); zzaba@ump.edu.pl (Z.Ż.); 6 Department of Forensic Medicine, Poznań University of Medical Sciences, 10 Rokietnicka Street, 60-806 Poznań, Poland; atezyk@ump.edu.pl (A.T.); czaba@ump.edu.pl (C.Ż.); 7 Department of Basic and Preclinical Sciences, Institute of Veterinary Medicine, Nicolaus Copernicus University in Torun, 87-100 Torun, Poland; 8 Department of Stem Cells and Regenerative Medicine, Institute of Natural Fibres and Medicinal Plants, Kolejowa 2, 62-064 Plewiska, Poland

**Keywords:** inflammation, psychotropic and antiepileptic drugs, biomarkers, anti-inflammatory treatment

## Abstract

In recent years, an increasing amount of research has investigated the impact of chronic inflammation on the development and progression of both neurological and psychiatric disorders, including epilepsy, depression, schizophrenia, and bipolar disorder. Moreover, growing attention is being paid to how inflammatory processes contribute to disease mechanisms, influence symptom severity, and interact with pharmacological treatments in these conditions. Changes in the levels of inflammatory biomarkers, such as cytokines and C-reactive protein, may signal the early stages of neurological disorder development. Furthermore, specific biomarker profiles have been identified for individual diseases, and chronic treatment may affect their blood levels. Over the last two decades, significant progress in the study of inflammatory biomarkers in psychiatric disorders and epilepsy has been achieved, demonstrating an association between biomarkers with symptoms, a potential prognostic role, and possible use in personalising therapy. Furthermore, widely used methods for biomarker evaluation, such as immunoenzymatic assays and flow cytometry, remain essential tools for current research. Despite numerous indications of the importance of inflammation in psychiatry and neurology, the available studies are characterised by considerable heterogeneity in terms of both population selection and methodology. Based on the available data, inflammatory biomarkers represent a promising diagnostic and therapeutic tool for epilepsy and psychiatric disorders. Although existing studies suggest a correlation between inflammation and the symptoms of various disorders, inconsistent results highlight the need for further research to enable wider implementation of these findings in psychiatric and epilepsy practice. Advancing knowledge of inflammatory biomarkers is essential for improving treatment outcomes and promoting the development of targeted interventions.

## 1. Introduction

While inflammation plays a crucial role in the body’s defence mechanisms, chronic activation of inflammatory processes can lead to severe dysfunction, including neuroimmunological disorders. Recent interest in the role of inflammation in epilepsy and psychiatric disorders suggests a link to immune dysfunction, the dysregulation of which may be a component of the pathogenesis of these conditions [1,2,3,4,5,6].

Inflammation is a complex physiological response to a variety of stimuli that is critical for maintaining health and homeostasis [1]. It involves immune cells and molecules, as well as vascular changes, which work together to repair tissue and eliminate noxious stimuli. Cytokines, chemokines and growth factors are the primary mediators of this process, modulating the activity of immune cells and promoting a balanced inflammatory response [7]. Traditionally, inflammation has been defined as a response to tissue damage and infection. However, the concept of inflammation has been broadened to include a wider range of conditions. The boundary between normal adaptive homeostatic adjustments and inflammation remains unclear [8]. Although inflammation is critical for defence and repair, it must be regulated, as chronic inflammation can lead to tissue damage and diseases [1,9].

Mental disorders such as depression, schizophrenia, and anxiety are a major global health problem. According to the World Health Organization (WHO), one in eight people worldwide were suffering from a mental disorder in 2022. Furthermore, there are predictions that the global burden of disease will increase [10]. Despite the considerable progress that has been made in understanding the pathogenesis of these disorders, their biological mechanisms remain incompletely understood. Researchers are becoming increasingly interested in how the nervous and immune systems interact, particularly with regard to the role of chronic inflammation in the etiology and progression of psychiatric disorders [2,11,12]. Epilepsy is also one of the most common neurological conditions, affecting over 70 million people worldwide. In patients with epilepsy, there are disorders in the humoral and cellular response, which are also affected by antiepileptic drug therapy. Immunological mechanisms may be responsible for the pathogenesis of some epileptic seizures. The possibility of immunoglobulin-based treatment in some people with epileptic seizures resistant to antiepileptic drug therapy is also important [5,13].

The relationship between inflammation and mental health is bidirectional, with inflammation potentially increasing the risk of epilepsy and psychiatric disorders and vice versa. Emerging evidence suggests a mutual interaction, whereby inflammation may contribute to their development and progression, and conversely, both disorders may also promote inflammatory responses [2]. Immune dysfunction is both a cause and an early indicator of the development of psychiatric and neurological disorders, where biomarkers have been correlated with immune dysfunction and the psychiatric disorders [14,15,16]. Researchers are also focusing on chronic psychiatric treatments that may induce inflammation or have anti-inflammatory effects, suggesting that it is possible to predict treatment efficacy or the development of new therapies for psychiatric disorders by analysing changes in levels of specific inflammatory biomarkers [17,18].

The most commonly studied biomarkers include cytokines such as interleukin 6 (IL-6), IL-1β, IL-4, IL-10 tumour necrosis factor alpha (TNF-α), interferon gamma (INF-γ), as well as C-reactive protein (CRP). These alterations are not only detected during acute episodes of the disease, but also in some patients during periods of remission, which may indicate a persistent inflammatory process of pathogenetic and prognostic significance. No clear pattern of immunomodulation has emerged to date, confirming that changes occur as a result of complex interactions [18,19,20].

A variety of analytical approaches are employed in inflammatory biomarker studies. The approach depends on various factors, including the study’s purpose, the number of parameters to be analysed, technological availability, and the sensitivity and specificity requirements of the assays. Selecting appropriate biological material that reflects the organism’s physiological or pathological state is also crucial.

Understanding the most common and frequently utilised markers for assessing inflammation in psychiatric conditions is crucial for advancing diagnostic precision, therapeutic strategies, and our fundamental understanding of these complex illnesses. Examining changes in inflammatory marker levels can deepen our understanding of how systemic immune signals impact brain function. Investigating inflammatory biomarkers is a promising way to identify potential therapeutic targets and develop personalised treatment approaches that address the specific inflammatory profiles of individual patients [19,21].

This review aims to summarises the current literature on inflammation in the context of epilepsy and selected psychiatric disorders—including depression, schizophrenia, and bipolar disorder—with particular focus on the biomarkers used and the analytical methods employed to determine them. The article not only synthesises the findings to date, but also identifies potential avenues for further research into the use of inflammatory biomarkers in psychiatry and neurology.

## 2. Analytical Biomarkers of Inflammation—Characteristics and Biological Functions

Cytokines, a diverse group of signalling proteins, are key to assessing inflammation in psychiatric disorders. These molecules, including interleukins, TNF-α and interferons, facilitate communication between immune cells and regulate neuronal function, neurotransmitter metabolism and neuroplasticity. The alterations in cytokines can be influenced by various factors, such as the duration of treatment, the specific antipsychotic drugs used, and the patient’s condition [22].

Understanding the role of inflammation requires a comprehensive overview of the most commonly used inflammatory markers in psychiatric research, including their functional roles and interactions with psychotropic drugs.

### 2.1. C-Reactive Protein

CRP is one of the most widely used markers of inflammation in medicine. It is an acute-phase protein, which is produced mainly by liver cells in response to inflammatory cytokines, particularly interleukin-6, as well as IL-1β and TNF-α. Its levels rise rapidly in response to infection, injury and other inflammatory stimuli—up to 1000-fold in a few hours—making it a highly sensitive, but non-specific, marker of the body’s inflammatory responses [23,24]. CRP is easily detectable in serum and plasma samples, which makes it useful for assessing the presence and severity of inflammation in clinical practice. Importantly, the protein has two distinct isoforms: pentameric CRP (pCRP), which is synthesised in the liver and exhibits the classical properties of CRP in circulation; and monomeric CRP (mCRP), which forms locally at sites of tissue injury and has stronger pro-inflammatory properties. The presence of these two forms may explain the systemic and local effects of CRP in response to different types of stimuli [23,25]. In the field of psychiatry, an increasing body of research suggests a link between elevated CRP levels and psychiatric disorders such as depression, schizophrenia and bipolar disorder. Chronic inflammation, as indicated by elevated CRP levels, may contribute to the pathophysiology of these conditions and influence the response to treatment. It may also have prognostic value, with higher levels being associated with greater symptom severity and a poorer therapeutic prognosis [24,26]. However, CRP levels are also affected by factors not directly related to the inflammatory process, such as obesity, smoking and stress. This can make interpreting the results difficult [23,25]. Nevertheless, increasingly accurate assays such as high-sensitivity CRP (hs-CRP) are making this marker a more important research tool and potential support for the diagnosis and treatment of psychiatric disorders [24].

### 2.2. Interleukin 6

IL-6 is one of the most well-researched cytokines and plays a key role in the body’s inflammatory, immune, and metabolic responses. It is a protein produced by a wide range of cells, including macrophages, monocytes, endothelial cells, fibroblasts, adipocytes and cells of the nervous system. While IL-6 has traditionally been viewed as a pro-inflammatory cytokine, an increasing amount of research challenges this concept, presenting IL-6 as a molecule with complex, dual biological activity [23,27]. IL-6 acts through two main signalling pathways: the classical pathway, which is activated by lower cytokine concentrations and is restricted to certain cells (e.g., hepatocytes), and the trans-signalling pathway, which uses the soluble IL-6R receptor to allow IL-6 to affect a wider range of cells. The classical pathway is mainly responsible for homeostatic functions such as the production of acute-phase proteins (e.g., CRP) or the regulation of metabolism. Trans-signalling, on the other hand, activates pro-inflammatory and pathogenic mechanisms, particularly in tissues without their own IL-6 receptors [23]. Through these two pathways, IL-6 can exert both pro-inflammatory functions, such as the recruitment of immune cells and the stimulation of fever, and anti-inflammatory functions by suppressing other inflammatory cytokines, such as IL-1β and TNF-α, and by promoting regenerative and repair processes [23,28]. IL-6 has attracted a lot of attention as a biomarker of low-grade chronic inflammation, which may be involved in the development of psychiatric disorders. Elevated levels of IL-6 have been observed in patients with major depression or schizophrenia. These levels correlate with symptom severity and a poorer response to pharmacological treatment [21,27,29]. However, moderate increases in IL-6 do not necessarily indicate inflammation. Rather, they may indicate the activation of mechanisms such as tissue repair, the maintenance of homeostasis, or the preparation of the immune system for potential threats. From this perspective, IL-6 acts as an indicator of the body’s commitment to maintaining biological integrity in response to various stimuli, rather than solely as a marker of pathological inflammation [23,28]. Its high dynamic range of concentration changes and its ability to reflect both acute and chronic physiological states make it a useful indicator of inflammatory processes. However, its multipotential effect makes interpreting the results challenging—an increase in IL-6 levels may indicate active inflammation, tissue repair or a response to exercise. Furthermore, most available assays do not distinguish between the activation of the classical and trans-signalling IL-6 pathways, which could significantly enhance the clinical value of the measurement [23].

### 2.3. Interleukin 1β

IL-1β is a crucial pro-inflammatory cytokine within the IL-1 family that plays a key role in the initiation and maintenance of inflammation. It is primarily synthesised by monocytes, macrophages, microglia, endothelial cells and astrocytes in the form of an inactive precursor known as pro-IL-1β. Unlike other cytokines, IL-1β requires a secondary signal, typically triggered by tissue damage or pathogens, to activate the inflammasome. Pro-IL-1β then undergoes proteolysis with caspase-1 to convert into its active form. IL-1β is a potent inflammatory mediator, inducing fever, stimulating the expression of adhesion molecules on endothelial cells, promoting the production of other cytokines such as IL-6 and TNF-α, and activating lymphocytes and macrophages. Within the nervous system, IL-1β influences neurogenesis, synaptic function and brain plasticity. It can cross the blood–brain barrier or be produced by activated microglia, demonstrating its impact on the function of the central nervous system [30]. IL-1β can be activated locally in neural tissue. Determining its concentration in cerebrospinal fluid (CSF) could provide more direct information about inflammation within the central nervous system. However, studies comparing IL-1β concentrations in CSF and peripheral blood have produced inconclusive results. A meta-analysis found no significant differences in IL-1β levels between CSF and peripheral blood in patients with psychiatric disorders [31]. Additionally, there are several limitations to measuring IL-1β levels. Its concentration in peripheral blood is usually very low, often falling below the detection sensitivity of immunological tests. IL-1β is locally active and rapidly degraded. Furthermore, most commercial tests fail to differentiate between the precursor form and the active cytokine, which can lead to the results being misinterpreted. Therefore, advanced methods such as an chemiluminescence immunoassay are necessary [32,33].

### 2.4. Interleukin 4 and 10

The interleukins IL-4 and IL-10 are anti-inflammatory cytokines that play an important role in regulating the immune response, maintaining homeostasis, and preventing the immune system from becoming overly activated. IL-4 is a small protein primarily produced by type 2 helper T cells (Th2), mast cells and basophils. IL-10, on the other hand, is synthesised by various cell types, including regulatory T lymphocytes (Treg), monocytes, dendritic cells and macrophages. Both cytokines act through specific membrane receptors, IL-4R and IL-10R, whose activation inhibits pro-inflammatory pathways. This includes the inhibition of nuclear factor kappa B (NF-κB) activation and the production of cytokines such as IL-1β, IL-6 and TNF-α. IL-4 is a major promoter of the humoral response. It stimulates B-lymphocyte differentiation and immunoglobulin class switching, especially to immunoglobulin E (IgE), while inhibiting macrophage and type 1 helper T cells (Th1) cytokine activity. IL-4 also has neuroprotective effects, reducing microglia activation and oxidative stress. IL-10 is a potent inhibitor of the inflammatory response; it inhibits antigen presentation, macrophage activation, and the production of pro-inflammatory cytokines [34]. Disturbed levels of these anti-inflammatory interleukins may promote chronic inflammation, making them interesting targets for future therapies and potential biomarkers of immune balance in psychiatry. Their levels may correlate with symptoms of psychiatric disorders, as has been observed in psychosis [35]. Furthermore, it has been demonstrated that IL-10 can modulate mesolimbic activity and dopamine release, making it a potential target for biological therapies in the treatment of inflammatory depression [36,37].

### 2.5. Tumour Necrosis Factor Alpha

TNF-α is a pro-inflammatory cytokine belonging to the TNF superfamily. It exists in both membrane-bound and soluble, biologically active forms. The latter is formed by the proteolytic action of the enzyme TACE (tumour necrosis factor-α converting enzyme). TNF-α is primarily produced by macrophages, monocytes, T cells and microglia in the central nervous system. It plays a central role in regulating the inflammatory response, cellular immunity and apoptosis. It stimulates the expression of adhesion molecules, activates neutrophils and monocytes, and stimulates the production of other cytokines, such as IL-1 and IL-6. TNF-α also influences oxidative stress and apoptosis pathways. In the nervous system, TNF-α modulates neurotransmitter function, blood–brain barrier permeability, and microglia activity. While it is involved in the body’s defence mechanisms, its overproduction can lead to tissue damage and chronic inflammation. This cytokine can interfere with serotonin metabolism, increasing extracellular glutamate concentrations and leading to overstimulation of glutamate (NMDA) receptors. This makes TNF-α a potential pathophysiological component of depressive, anxiety-related and cognitive symptoms. Furthermore, these changes may contribute to treatment resistance [38,39].

## 3. Analytical Determination of Inflammatory Biomarkers

Cytokines are among the most commonly measured markers for psychiatric disorders and psychotropic medication. In practice, however, accurate detection of cytokines is difficult due to the significant impact of pre-analytical and analytical errors. Biomarker analysis involves applying a variety of methods to different biological materials, each with its advantages and disadvantages. The most commonly used materials are blood (serum and plasma), cerebrospinal fluid, urine and saliva. Even neuroimaging techniques can be used to identify biomarkers [40]. Blood is a rich source of circulating proteins, metabolites and genetic material, and is readily available and amenable to repeated sampling [41]. However, for alternative matrices such as CSF, saliva and in vitro supernatant, it should be noted that their concentrations are not correlated with systemic levels of the same cytokines [42].

Cytokines typically have a short half-life, sometimes to the order of minutes. The time between sample collection and processing can significantly impact their measured levels [32,41]. Additionally, cytokines are present in the body in trace amounts, which makes them challenging to detect and accurately quantify. Cytokine detection can also be markedly affected by how biological samples are handled throughout the entire process, from initial sampling to laboratory analysis [32]. Inconsistent sample collection, processing and storage can result in inaccurate measurements [41]. For instance, the stability of cytokines can fluctuate in unprocessed blood under varying storage conditions. Accurate interpretation requires sample collection and handling procedures to be reported alongside quantification data [32].

According to an analysis of conducted studies, the methods most commonly used for quantitative detection when assessing biomarkers for psychiatric disorders are classical ELISA immunoassays, Luminex-type multiparameter techniques and flow cytometry. Blood, either plasma or serum, is the main medium used. Studies evaluating the in vitro effects of psychotropic drugs on changes in cytokine concentrations also use whole blood, specifically the cell-free culture supernatant of stimulated whole blood. A comparative summary of the most commonly used analytical techniques and biological materials for each biomarker is shown in Table 1.

## 4. Psychotropic and Antiepileptic Drugs and Inflammatory Biomarkers: An Immunological Perspective of the Mutual Relationship

Depression, schizophrenia, anxiety disorders and bipolar disorder with epilepsy are among the most commonly diagnosed mental health disorders, presenting significant challenges to modern psychiatry and neurology [10,13]. While they differ in their clinical presentation, progression and treatment, these disorders may involve common pathophysiological mechanisms, such as disturbances in the immune system and chronic inflammation. In recent years, there has been an increase in interest in the role of cytokines and other inflammatory biomarkers in the development and progression of psychiatric illnesses, as well as in the effects of drug treatment—including antipsychotics and antidepressants—on their concentrations [2,11,12,57]. In addition to the above disorders, epilepsy, although formally classified as a neurological disorder, is also worth considering. There is increasing evidence of a relationship between epilepsy and immunological changes which overlaps with observations in patients with psychiatric disorders. Chronic inflammation, elevated cytokine concentrations and the effects of antiepileptic drugs on the immune system suggest partly shared biological pathways. Furthermore, some antiepileptic drugs exhibit immunomodulatory properties that are comparable to those observed with antipsychotics, which further justifies their juxtaposition in the context of inflammatory biomarkers [47,51,58].

The primary type of treatment for psychiatric disorders is pharmacotherapy. Importantly, some drugs have applications in the treatment of more than one condition–for example, valproic acid is also sometimes used to treat bipolar disorder, and some psychotropic drugs are used as an additional treatment for drug-resistant depression. This therapeutic link between psychiatric and neurological disorders further highlights the importance of considering them together in the context of immunological changes and inflammatory markers [59,60].

The main drugs used to treat epilepsy are antiepileptic drugs. The most commonly used drugs are carbamazepine, sodium valproate, lamotrigine and levetiracetam. The drugs mainly act by modulating sodium and calcium channels, as well as GABA-ergic transmission [61]. Antiepileptics have attracted attention for their potential effects on serum inflammatory factors and immune function, which may have implications for neuroinflammation and therapeutic strategies in neurological disorders [58]. One of the most commonly used antiepileptic drugs is valproic acid (VPA). This drug, which is a first-line treatment, is characterised by its broad spectrum of action and its use in many neurological and psychiatric disorders [62]. Several studies have investigated the effects of valproate on inflammatory markers and immune function, with mixed results. Some studies reported reduced cytokine levels after VPA treatment, while others found no significant changes [58]. A study in healthy male subjects found a significant increase in plasma IL-6 levels after one week of valproate treatment (1000 mg/day). There was also a positive correlation between plasma VPA levels and changes in plasma IL-6. These findings suggest that VPA may have a modulating effect on the pro-inflammatory cytokine IL-6 in humans [46]. In contrast, studies in epileptic patients have shown that chronic VPA treatment does not cause significant differences in IL-6 and IL-1β levels [47,48,55]. Research conducted by Sonmez et al. showed that 12 months of VPA therapy in children resulted in a significant increase in pro-inflammatory IL-1α and a decrease in anti-inflammatory IL-10 [47]. Findings from rat models suggest a possible neuroprotective, including anti-inflammatory, effect of VPA. This may be due to a reduction in glial cell activation in the brain and the release of pro-inflammatory factors [63].

A relatively new antiepileptic drug is levetiracetam (LEV), which is a second-generation AED with better tolerability and higher efficacy than other antiepileptic drugs. In contrast to VPA, which mainly blocks sodium channels, LEV interacts with synaptic vesicle protein 2A (SV2A), thereby affecting neurotransmitter release. It can also affect neuronal calcium levels either by blocking voltage-gated Ca^2+^ channels or by inhibiting intracellular calcium release [64]. Despite limited clinical evidence of its anti-inflammatory effects, comparative studies of LEV and VPA—as old and new AEDs—showed no significant changes in IL-6, IL-1β and TNF-α levels in patients receiving VPA and LEV monotherapy and VPA+LEV combination treatment [48,55]. However, studies in children with epilepsy have shown a significant decrease in C–C Motif Ligand 2 (CCL2). This reduction was greater in patients treated with VPA+LEV than in those treated with VPA alone [55]. In contrast to the above studies on chronic use of the drug, studies focusing on maximum serum LEV concentrations after drug administration showed an effect on serum IL-1β levels. Comparisons of drug concentrations (pre-drug; 1, 2, 4 and 8 h post-drug) and IL-1β levels showed a statistically significant decrease in pro-inflammatory interleukin levels, which was negatively correlated with drug concentrations. However, the authors emphasise that these studies did not include long-term drug use, which may indicate that LEV only had an effect on the interleukin of interest for a short period of time after the maximum serum concentration was reached [56].

Lamotrigine (LTG), like LEV, is a second-generation AED, but like VPA it is also a voltage-gated sodium channel blocker. The studies carried out have mainly focused on comparing this drug with other AEDs, mainly VPA. The results obtained by Du et al. indicate that there was a statistically significant difference in IL-1β, IL-2, IL-6 and TNF-α levels after six months of LTG therapy. Based on the data, they concluded that LTG may inhibit the secretion of pro-inflammatory cytokines. Furthermore, this difference was greater than in patients treated with VPA [65]. Evaluation of the in vitro immunological properties of the drugs using the toxic shock syndrome toxin (TSST-1) also showed that AEDs can modulate the signalling pathways of selected cytokines. For IL-1β, IL-2 and TNF-α, no difference was observed between LEV and LTG. Both drugs significantly reduced the levels of the markers tested. Inconsistencies were found for IL-22, where LEV, VPA and LTG caused changes in different directions. These were, respectively, an increase, a decrease and no change in IL-22 concentrations [51].

The antiepileptic mechanism of action involving sodium channels includes not only VPA, but also carbamazepine (CBZ) and oxcarbazepine (OXC). The second drug belongs to the second generation of AEDs and was synthesised as a potential successor to the first-generation drug CBZ. Although these drugs have a similar chemical structure, they differ, among other things, in their metabolic pathways and side effects [66,67]. Himmerich et al. demonstrated the same effect of CBZ and OXC on IL-22 as LEV. The changes in IL-1β and IL-2 levels induced by CBZ were comparable to those observed with the second-generation drugs LEV and LTG [51]. A similar effect on pro-inflammatory interleukins was observed during the evaluation of CBZ’s anticonvulsant activity in rats. The 14-day treatment resulted in a decrease in the levels of not only IL-1 but also of IL-6 [68].

In the treatment of schizophrenia, the main group of drugs are antipsychotics. As with AEDs, there are several generations of antipsychotics and a variety of mechanisms of action. However, they are associated with a variety of side effects, including those on the immune system [2,69,70]. First-generation antipsychotics such as haloperidol (HLP) or chlorpromazine (CHPZ) are known for their dopaminergic antagonism, which effectively reduces the symptoms of psychosis [69]. Previous studies suggest that HLP may cause a dose-dependent reduction in the levels of the pro-inflammatory cytokines IL-6 and TNF-α [71], which is supported by a recent study that demonstrated a reduction inIL-6 and cortisol concentrations [72]. Studies in rats indicate that HLP causes a number of biological alterations and can cause a reduction in the activity of the complement system [73].

The effect of drug dose and, in addition, the degree of glial cell activation on the immune system was also confirmed by Obuchowicz et al. [71]. Slightly activated glial cultures exposed to LPS, HLP (0.5, 5; 10 μM), risperidone (RIS) (5; 10 μM) and CHPZ (10 μM) shifted the balance between the cytokines towards the anti-inflammatory. In contrast, HLP and RIS had a negative immunoregulatory effect in highly activated glial cells. Interestingly, this was only observed at their highest concentrations. They caused an increase in IL-1β and TNF-α and a decrease in IL-10 levels. At all concentrations, the effect of CHPZ on IL-10 levels was weaker than that of RIS and HLP [74]. CHPZ, but not HLP, also showed a different effect on cytokines compared with the second-generation drugs. The drug led to an increase in TNF-α and IL-2 levels when blood TSST-1 was stimulated, in contrast to the atypical drugs (quetiapine and N-desmethylclozapine), which led to an increase. Consistent results were obtained for IL-6 and IL-1β, for which no significant changes were observed for any drug at any of the concentrations tested. In addition, there was an increase in IL-4 concentrations, and it was the highest for CHPZ [75].

Atypical antipsychotics appear to have a different effect on the immune system because of their different mechanisms of action and lower frequency of side effects, such as neuroleptic malignant syndrome or extrapyramidal symptoms [76,77,78]. However, as with classical antipsychotics, the effect on the cytokine profile is complex and not well defined. Differences exist not only between studies of different representatives of this group, but also between studies of the same substance. Risperidone has been shown to reduce the levels of TNF-α, IL-10, IL-13 and IL-17α [54,79]. Similarly, its use may lead to a decrease in IL-4 [54]. In contrast, conclusions from a meta-analysis suggest that the drug has a pro-inflammatory effect by increasing IL-6 concentrations [80]. Gender differences have been shown for clozapine. In both men and women, chronic use of this drug caused abnormal expression of IL-2, IL-6, IL-17 and TNF-α. However, the increase in IL-2 and IL-1β was lower in women. In addition, IL-2 showed a significant positive correlation between its concentration and drug dose, which occurred only in women [81].

The effects of individual drugs on cytokine levels are shown in Table 2.

The complex relationship between inflammation and psychiatric disorders offers significant opportunities for the development of new treatments. Given the evidence supporting the role of inflammation in mental health disorders, anti-inflammatory treatments have been considered as additional or primary therapeutic strategies. Conducted research has highlighted the potential advantages of various anti-inflammatory agents. However, the integration of anti-inflammatory treatments into standard psychiatric care is responsible for challenges, in particular the need for a robust understanding of individual differences in inflammatory responses and potential side effects associated with chronic anti-inflammatory use [82,83].

The heterogeneity of psychiatric and neurological disorders complicates the understanding of the role of the immune system, leading to inconsistent results between studies. In addition, the potential for long-term side effects related to anti-inflammatory drugs raises concerns about their safety. The pathways by which inflammation influences brain function remain poorly understood, requiring additional exploration of the mechanisms underlying these effects. The potential benefits of anti-inflammatory treatments in psychiatric disorders are underlined by findings of various clinical trials. A remarkable example is the use of non-steroid anti-inflammatory drugs (NSAIDs) and cytokine inhibitors [84]. Anti-inflammatory therapies, particularly cyclooxygenase-2 (COX-2) inhibitors such as celecoxib, have shown promising results. Studies have shown that adjuvant treatment with celecoxib improves symptoms of psychiatric disorders compared with antidepressants alone. The efficacy appears most pronounced in patients with higher baseline inflammatory markers [84,85,86,87].

When considering drugs used as monotherapy or adjunctive treatment for the treatment of psychiatric disorders, a distinction can be made not only between agents that have a primary anti-inflammatory effect, but also those that have additional anti-inflammatory properties in addition to their primary action. Table 3 shows examples of the drugs most commonly used to reduce inflammation in psychiatric disorders [20,82,84,88].

## 5. Correlation Cycle—Inflammation, Psychiatric and Neurological Disorders with Psychotropics and Antiepileptics

**Figure 1:**
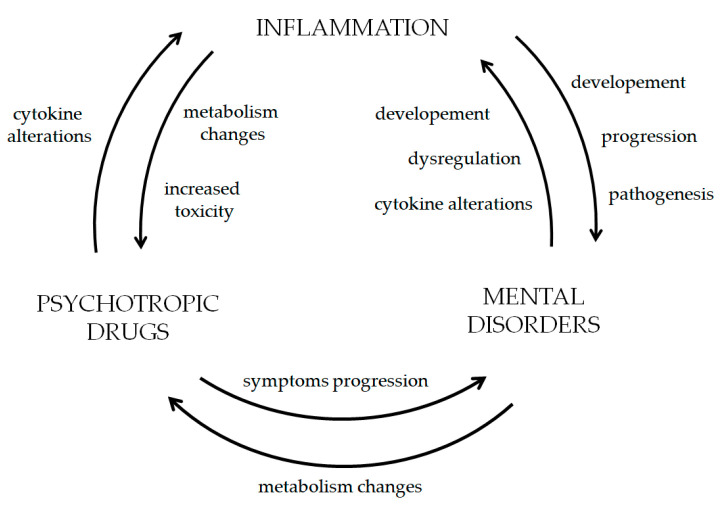
Cycle of inflammation and mental disorders [2,89,90].

The relationship between inflammation and psychiatric and neurological disorders such as epilepsy is characterised by an inseparable, interdependent relationship in which each one influences the other, creating a perpetual cycle, as shown in Figure 1. In addition, the psychotropic and antiepileptic drugs used to treat these disorders can influence the inflammatory state, creating a cyclical relationship characterised by constant feedback loops whose exact starting point is unknown [2,89,90].

Antipsychotic and antiepileptic drugs have demonstrated varying effects on inflammatory markers in both clinical and experimental studies. In vivo studies often show high levels of pro-inflammatory cytokines in patients with mental illnesses such as depression and schizophrenia. Baumeister et al. suggested that cytokine levels may serve as biological markers for diagnosing and understanding psychiatric disorders, supporting the hypothesis that systemic inflammation plays a key role in the pathophysiology of these diseases [19]. In vitro research conducted by Obuchowicz et al. demonstrated that antipsychotic drugs have the capacity to modulate the immune response by affecting the balance between pro- and anti-inflammatory cytokines. The effects of these drugs appear to be concentration-dependent and vary based on the level of glial cell activation [74]. Other studies reported both pro- and anti-inflammatory activity, even for the same antipsychotic drug. Differences were also observed between drugs in a given group, such as the antidepressants clomipramine and fluoxetine, which reduce levels of IL-6, TNF-α and interferon γ (INF-γ), whereas others, such as mirtazapine and venlafaxine, tend to increase cytokines [19].

A meta-analysis of second-generation antipsychotics (risperidone and clozapine) and inflammatory cytokine levels in schizophrenia patients shows the complex interplay between treatment, inflammation and disease. The observed differences in their effects on inflammatory markers emphasise the heterogeneity of antipsychotic drugs, even within the same generation. Furthermore, variations in cytokine levels between patients with chronic schizophrenia and those experiencing their first episode of psychosis imply that illness duration also influences the inflammatory response to antipsychotic treatment [22].

The relationship between psychotropic drugs treatment and increased cytokine levels could also be related to metabolic side effects like weight gain. Risperidone increases appetite and overeating, leading to weight gain, which contributes to increased inflammatory markers [80].

This issue concerns not only drugs used in therapeutic doses, but also acute intoxication with psychotropic drugs. Studies investigating the effects of quetiapine overdose on oxidative stress and inflammatory markers showed increased levels of pro-inflammatory cytokines such as TNF-α and IL-6. This was also accompanied by an increase in the levels of markers of oxidative stress. Furthermore, C-reactive protein (CRP) levels were positively correlated with the occurrence of tachycardia. Despite the lack of research on the toxic concentrations of psychotropic drugs, these results suggest that overdose can lead to elevated levels of pro-inflammatory cytokines, resulting in the activation of inflammatory responses [44].

Circular dependence describes the dynamic interaction between drugs and biomarkers, illustrating the relationship between each component continually influencing the other components. Therefore, circular dependence highlights the need for an integrated therapeutic approach that addresses psychiatric and inflammatory factors to promote better mental health outcomes.

## 6. Discussion

The correlation between the immune system and psychiatric/neurological disorders has been addressed in this manuscript. However, the complexity of the topic limits the scope of discussion in this manuscript. Changes in inflammatory marker levels have been observed in patients with these disorders [1,2,3,4]. In particular, elevated levels of cytokines such as IL-6, IL-1β, IL-4, IL-10, TNF-α and CRP are frequently reported. The direction and intensity of these changes depend on various factors, including the type of disease and the type and duration of drugs used. These biomarkers have been shown to correlate with the severity of psychiatric symptoms, as well as with the response to treatment, suggesting their potential for prognosis. An imbalance between the activation and inhibition of the inflammatory response may play an important role in the pathogenesis of psychiatric disorders and affect response to treatment [2,18,19,22,91].

Nevertheless, it is important to note that levels of inflammatory biomarkers are affected not only by the mental health condition itself, but also by the pharmacotherapy used to treat it. For example, antidepressants and antipsychotics have different immunomodulatory effects. Even within the same drug group, different effects on the immune system have been observed. The intensity of drugs modulating the immune response also depends on the duration of therapy and the dose. Therefore, when interpreting the results of inflammatory biomarker determinations, it is important to consider not only the clinical picture, but also the context of pharmacotherapy, which poses an additional methodological challenge [19,74].

From a clinical perspective, the use of inflammatory biomarkers could, in the future, enable the identification of inflammatory subtypes of depression or schizophrenia, enabling personalised treatment. There is already a growing interest in using immunomodulatory drugs, such as infliximab and celecoxib, to treat drug-resistant depression and other psychiatric disorders, particularly in patients with elevated baseline inflammatory markers. Preliminary clinical trial results suggest that anti-inflammatory interventions may alleviate depressive symptoms, particularly when chronic, low-grade inflammation is present. Although anti-inflammatory treatments are not yet part of the therapeutic standard, they are increasingly being considered as an adjunct to psychotropic therapy for selected patient groups [17,18].

A major challenge in using anti-inflammatory treatments for mental health disorders is the need to tailor treatment to different types of inflammation. Mental health disorders can have different patterns of inflammation, so treatment needs to be carefully matched to these differences. Current research shows that not all people with mental health problems have high levels of the same inflammatory markers. Therefore, the same anti-inflammatory treatments may not be effective for everyone and could even be harmful in some cases. Identifying and grouping different types of inflammation could help target treatments more precisely [92,93]. On the other hand, patients with elevated inflammatory markers may respond better to additional anti-inflammatory strategies. The complexity of inflammatory patterns suggests that personalised treatment approaches may optimise therapeutic outcomes [94].

The potential of anti-inflammatory treatments must be approached with caution, as recent research shows conflicting results. Du et al. showed that anti-inflammatory monotherapy was no better than a placebo. However, when anti-inflammatories were combined with traditional antidepressants, significant improvements were noted. The potential of anti-inflammatory treatments may depend on how they are integrated with existing therapies [65].

Although inflammatory biomarkers appear to be a promising tool for supporting diagnosis and personalising psychiatric therapy, their practical application remains limited. The available literature is characterised by a high heterogeneity in terms of study design, patient population, diagnostic criteria, and biomarker determination methods. Standardised reference values for inflammatory markers in the psychiatric context are still lacking, as is data enabling clinical interpretation of results. Another significant problem is the difficulty of determining their presence in biological material. Time is a particularly important factor affecting the final result. This is not only because cytokines have a short half-life, but also because of the influence of sample processing time and storage conditions [32,41]. Untreated blood samples whose plasma/serum has been in contact with cellular elements have been shown to increase IL-6 and TNF-α levels [41]. Another limitation is the very low concentrations of cytokines, which require suitably sensitive methods. Furthermore, real-time analysis is challenging due to the complexity of the cytokine network, which exhibits multiple synergistic and antagonistic mechanisms [32]. One of the most commonly used methods for determining the presence of inflammatory markers is ELISA, which enables quantitative analysis of single analytes. In addition, multiplex immunoassays allow the simultaneous determination of dozens of analytes in small sample volumes, significantly reducing the analysis time. A limitation of these methods is the inability to distinguish between active and inactive forms of certain cytokines (e.g., IL-1β), which may result in an incomplete assessment of their biological activity [32,41].

One of the most commonly used methods for determining inflammatory markers is the enzyme-linked immunosorbent assay (ELISA), which enables quantitative analysis of single analytes. In addition, multiplex immunoassays, based on bead- or array-based technologies, allow the simultaneous determination of dozens of analytes in small sample volumes, significantly reducing analysis time. Modern multiplex platforms are optimised to minimise potential cross-reactivity.

In most studies of inflammatory markers in psychiatry, the biological material used for analysis is blood, primarily serum or plasma, and in vitro, whole blood. These samples are relatively easy to obtain and store, which facilitates their use in clinical studies. Nevertheless, in recent years, there has been growing interest in alternative matrices, such as saliva and CSF. While some of these allow non-invasive or more specific access to certain biological systems, they are still not widely used. The main issue is the lack of well-documented correlations between marker levels in these alternative materials and their concentrations in serum or plasma. Additionally, the lack of standardisation in collection procedures, storage, and interpretation of results makes it difficult to compare data between studies, limiting their clinical utility [42,95,96].

Another methodological point to note in these studies is the study model: in vitro versus in vivo. This has a direct impact on the results of inflammatory biomarkers. Furthermore, differences between the results of the two models have been demonstrated. While cellular models allow detailed tracking of the molecular mechanisms and effects of specific cytokines or drugs, they do not always reflect the complexity of an organism’s biological environment. Under in vivo conditions, the inflammatory response is regulated by multiple interacting systems, such as stress, the diurnal rhythm and nutritional status. Many antipsychotics induce metabolic syndrome, which is associated with inflammation. It is therefore difficult to determine whether an increase in inflammatory markers is a direct result of the drug or a side effect. These differences highlight the importance of carefully interpreting experimental data and combining research models with clinical observations to gain a more comprehensive understanding of the role of inflammatory markers in psychiatric disorders [19].

## 7. Conclusions

Based on the available research findings, it can be concluded that psychiatric disorders are not only ‘neuro’ but also ‘immuno’ diseases. This makes them highly complex conditions, with the inflammatory response potentially playing a significant role in their pathophysiology [2]. Observed changes in cytokine levels can be a cause or an effect of psychiatric symptoms. Determining inflammatory markers could open a new ‘gateway’ to their diagnosis and treatment. However, it should be noted that interpreting biomarker data involves many variables that need to be taken into account, and there are still many unresolved issues.

The field of biomarkers related to psychiatric disorders is complicated, and future studies should include well-characterised patient groups that use sensitive, standardised analytical methods. Integrating inflammatory biomarkers with clinical data, neuroimaging, and other biological materials may contribute to the future development of more precise diagnostic and therapeutic tools in psychiatry. In parallel, it is beneficial to continue researching the effects of psychotropic drugs on the cytokine profile, as well as their potential immunomodulatory effects, both as side effects and deliberate therapeutic actions.

## Figures and Tables

**Table 1:** Techniques used to quantify selected inflammatory biomarkers.

Protein Designation	Analytical Methods	Biological Material	Patients/Evaluated Drug	Reference
CRP	ELISA	Whole blood	Healthy volunteers	[43]
	Immunoturbidimetry	Serum	Quetiapine overdose	[44]
		Serum/plasma	Epileptic patients; OXC	[45]
IL-6	ELISA	Plasma	Healthy male volunteers; VPA	[46]
		Serum	Epileptic children; VPA	[47]
			Epileptic patients; VPA, LEV	[48]
			Schizophrenic patients; RIS	[49]
	Flow cytometry	Whole blood	Epileptic patients; CBZ, LTG, VPA	[50]
			Healthy female volunteers; a.o LTG, CBZ, VPA, OXC, LEV	[51]
		Plasma	Epileptic patients; LTG, VPA	[52]
			Schizophrenic patients	[53]
		Serum	First-episode psychosis; RIS	[54]
IL-1β	ELISA	Serum	Epileptic children; VPA	[47]
			Schizophrenic patients; RIS	[49]
			Epileptic patients; VPA, LEV	[48]
			Epileptic children; VPA, LEV	[55]
	CLIA	Serum	Epileptic patients; LEV	[56]
	Flow cytometry	Whole blood	Healthy female volunteers; a.o LTG, CBZ, VPA, OXC, LEV	[51]
		Plasma	Epileptic patients; LTG, VPA	[52]
IL-4	Flow cytometry	Whole blood	Healthy female volunteers; a.o LTG, CBZ, VPA, OXC, LEV	[51]
		Serum	First-episode psychosis; RIS	[54]
		Plasma	Schizophrenic patients	[53]
IL-10	ELISA	Serum	Epileptic children; VPA	[47]
	Flow cytometry	Whole blood	Epileptic patients; CBZ, LTG, VPA	[50]
		Serum	First-episode psychosis; RIS	[54]
		Plasma	Schizophrenic patients	[53]
TNF-α	ELISA	Serum	Epileptic children; VPA	[47]
			Epileptic patients; VPA, LEV	[48]
			Schizophrenic patients; RIS	[49]
	Flow cytometry	Whole blood	Healthy female volunteers; a.o LTG, CBZ, VPA, OXC, LEV	[51]
		Serum	First-episode psychosis; RIS	[54]
		Plasma	Epileptic patients; LTG, VPA	[52]

Abbreviations: CRP—C-reactive protein; IL-6—interleukin 6; IL-1β—interleukin 1β; IL-4—interleukin 4; IL-10—interleukin 10; TNF-α—tumour necrosis factor-α; ELISA—enzyme-linked immunosorbent assay; CLIA—Chemiluminescent Immunoassay; OXC—oxcarbazepine; VPA—valproic acid; LEV—levetiracetam; RIS—risperidon; CBZ—carbamazepine; LTG—lamotrigine.

**Table 2:** Changes in cytokine levels during treatment with psychotropic and antiepileptic drugs.

	Antiepileptic Drugs			Psychotropic Drugs		
	Drug	Cytokine Changes	Reference	Drug	Cytokine Changes	Reference
First generation	VPA	↑ IL-6 −IL-6, IL-1β ↓ IL-22	[8] [47,48,55] [51]	HLP	↓ IL-6, TNF-α ↓ IL-6 ↓ IL-10 ↑ IL-1β, TNF-α	[71] [72] [74] [74]
	CBZ	↓ IL-22 ↓ IL-1, IL-6	[51] [68]	CHPZ	↑ TNF-α, IL-2 ↑ IL-4	[75] [75]
Second generation	LTG	↓ IL-6, IL-1β, IL-2, TNF-α −IL-22	[52] [51]	RIS	↓ IL-10 ↑ IL-1β, TNF-α ↓ TNF-α, IL-10 ↑ IL-4 ↑ IL-6	[74] [74] [54] [54] [80]
	LEV	−IL-6, IL-1β, TNF-α ↓ IL-1β ↑ IL-22	[48,55] [56] [51]	CLO	↑ IL-2, IL-6, TNF-α	[81]

Abbreviations: IL-1—interleukin 1; IL-1β—interleukin 1β; IL-2—interleukin 2; IL-4—interleukin 4; IL-6—interleukin 6; IL-10—interleukin 10; IL-22—interleukin 22; TNF-α—tumour necrosis factor-α; VPA—valproic acid; CBZ—carbamazepine; LTG—lamotrigine; LEV—levetiracetam; HLP—haloperidol; CHPZ—chlorpromazine; RIS—risperidon; CLO—clozapine; ↑—increase in concentration; ↓—decrease in concentration; (−)—no change in concentration.

**Table 3:** Anti-inflammatory drugs used in the treatment of psychiatric and neurological disorders [20,82,88].

Drug Category	Drug	Molecular Mechanism Used in Therapy
Cytokine inhibitors	Adalimumab	TNF-α inhibition
	Infliximab	
	Etanercept	
NSAIDs	Celecoxib	Non-selective COX-inhibition/Selective COX-2 inhibition
	Aspirin	
	Ibuprofen	
	Naproxen	
Antibiotics	Minocycline	Anti-inflammatory, antioxidant and neuroprotective effects on CNS
	Rapamycin	
Antidiabetic drugs	Pioglitazone	Anti-inflammatory, neuroprotective, and anti-excitotoxic effects
	Metformin	
Statins	Simvastatin,	Anti-inflammatory and antioxidant effects (NMDA receptor modulation and inhibition of NO)
	Atorvastatin	
	Lovastatin	

Abbreviations: TNF-α—tumour necrosis factor-α; NSAIDs—nonsteroidal anti-inflammatory drugs; COX—cyclooxygenase; COX-2—cyclooxygenase 2; CNS—central nervous system; NMDA—N-methyl-D-aspartate, NO—nitric oxide.
